# Why is there motor deterioration in Parkinson’s disease during systemic infections-a hypothetical view

**DOI:** 10.1038/npjparkd.2015.14

**Published:** 2015-08-27

**Authors:** Florian Brugger, Roberto Erro, Bettina Balint, Georg Kägi, Paolo Barone, Kailash P Bhatia

**Affiliations:** 1 Sobell Department of Motor Neuroscience and Movement Disorders, Institute of Neurology, University College of London, London, UK; 2 Department of Neurology, Kantonsspital St Gallen, St Gallen, Switzerland; 3 Centre of Neurodegenerative Diseases-CEMAND, University of Salerno, Salerno, Italy

## Abstract

Clinicians are well aware of the fact that patients with Parkinson’s disease may significantly deteriorate following a systemic infection or, in its most severe case, may even develop an akinetic crisis. Although this phenomenon is widely observed and has a major impact on the patients’ condition, the knowledge about the underlying mechanisms behind is still sparse. Possible explanations encompass changes in the pharmacodynamics of the dopaminergic drugs, altered dopamine metabolism in the brain, alterations in the dopaminergic transmission in the striatum or an enhancement of neurodegeneration due to remote effects of peripheral inflammatory processes or circulating bacterial toxins. This article provides possible explanatory concepts and may hence support formulating hypothesis for future studies in this field.

## Introduction

Parkinson’s disease (PD) is characterized by a progressive loss of dopaminergic neurons. The clinical picture encompasses motor and nonmotor symptoms with a slow deterioration over years.^1^ A considerable number of patients, however, may experience a subacute worsening of their condition including their motor symptoms during systemic infections.^2^ Not all patients completely recover from this deterioration, thus leading to a higher level of disability and the requirement of higher doses of dopaminergics afterwards.^2,3^ In its most severe form, infections may trigger a life-threatening akinetic crisis featuring severe akinesia, cognitive and psychotic disturbances, fever, impaired consciousness, dysphagia, and speech problems. Patients then often become unresponsive to antiparkinsonian drugs.^4^ Infections are furthermore often accompanied by delirium, in PD frequently presenting in its’ hypoactive form.^5^ Although the diagnosis of delirium defined as an acute organic brain disorder with cognitive impairment, attention disorders, reduced level of consciousness, altered psychomotor activity, and wake–sleep rhythm disorders does not necessarily require the presence of motor symptoms, its occurrence is a risk factor for both long-term motor and cognitive deterioration in PD.^6^ PD deterioration associated with infection is mostly noticed by clinicians when their patients are admitted to the hospital or, though less marked, when they come to the outpatient clinic.^3^ Hence, PD deterioration after infections may have a wide spectrum ranging from mild worsening to akinetic crisis as the most severe form.

**Figure 1:**
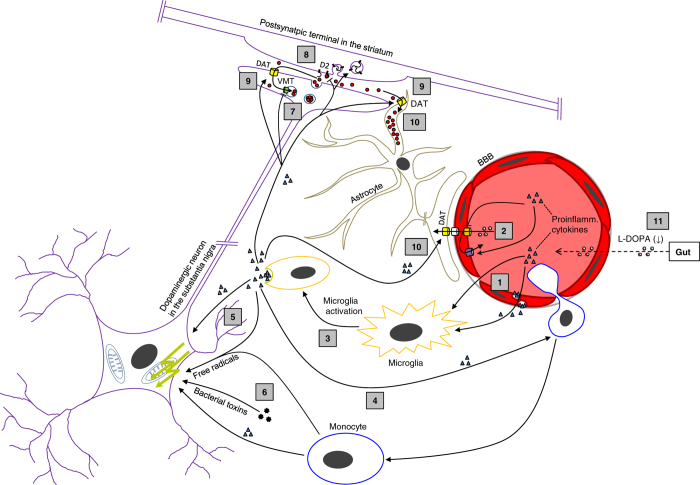
Proinflammtory cytokines (triangles) may diffuse into the brain through leaks in the blood–brain barrier (BBB) or may be transported through the BBB by endothelial transcytosis (1). At the BBB itself, cytokines may alter the transport of dopaminergic drugs (red–white circles) across the BBB by downregulating membrane transporters or by actively eliminating dopaminergic drugs (2). In the brain, proinflammatory cytokines may activate quiescent microglia (3) and may recruit circulating monocytes (4), an immune cell line closely related to microglia. Upon activation, microglia itself releases cytokines and reactive oxygen metabolites that in turn have a detrimental effect on neurons due to an increased level of oxidative stress and mitochondrial dysfunction (5). In addition, circulating bacterial toxins may also be capable to directly damage dopaminergic neurons (6). At the site of dopaminergic transmission, cytokines may impair the packaging of dopamine into vesicles (7) thus reducing the availability of dopamine at the presynaptic terminals and the synaptic cleft. Cytokines may also downregulate dopamine receptors (D2) at the postsynaptic terminals (8) and increase the reuptake of dopamine e.g., through upregulating the expression of DAT by neurons and nearby astrocytes (9). The increased reuptake, particularly into the astrocyte pool, may lead to a compartmentalization of dopamine (10). Another relevant factor for PD deterioration during systemic infections may be an altered medication intake resulting in a reduced availability of dopaminergics at the BBB and in the brain, respectively (11). DAT, dopamine active transporter; VMT, vesicular monoamine transporter.

The view of previous studies on this issue has often been blurred by focussing on reasons for subacute deterioration of PD as a whole, particularly in the context of hospitalizations.^7,8^ There is only one study by Umemura *et al.* who systematically analyzed the impact of systemic infections alone in a retrospective case–control study.^2^ Of 80 PD patients with systemic inflammation, 26 had experienced prolonged and sustained motor deterioration afterward. High body temperature and delirium were significantly associated with motor deterioration, whereas other factors like patient’s age, disease duration, pre-existing Hoehn and Yahr stage, and the presence of dementia were not predictive in this regard. Beyond this study, evidence is limited in this field. One major gap, besides the considerable impact of infections itself, is the lack of understanding of pathophysiological mechanisms underlying infection-related PD deterioration, underscoring the need for further studies. The aim of this viewpoint is to provide some pathophysiological considerations, which could be integrated for future studies. We have divided these under the following possibilities: (1) altered medication intake; (2) altered dopamine metabolism and receptor signaling; (3) enhancement of neurodegeneration by peripheral inflammation (Figure 1).

## Altered medication intake

Motor deterioration of PD during infections can often be explained by dosing errors or the administration of antidopaminergic drugs (e.g., antiemetics). Gerlach and colleagues recently in a prospective study analyzed the causes of PD patients’ deterioration during hospitalization. A considerable proportion of their patients were admitted to the hospital owing to infections and one of the main reasons for motor deterioration was altered and incorrect drug intake or the administration of antidopaminergic drugs.^8^ Altered cognition and psychosis, which can also be precipitated by infections in PD, also put patients at a higher risk of making dosing errors, particularly in the community, when they are often not supervised by a carer.^9^ In the case of dosing errors, one might assume that a mere treatment adjustment would be sufficient to improve motor symptoms, but this is not always observed after infections, which points to other possible relevant mechanisms.^2^ Intriguing insights in this regard may come from the dopamine agonist withdrawal syndrome (DAWS) and the neuroleptic malignant syndrome (NMS). DAWS is characterized by the occurrence of severe nonmotor symptoms such as psychiatric disturbance including anxiety, apathy and depression, nausea, hyperhidrosis, and pain following the decrease or cessation of dopamine receptor agonists,^10,11^ whereas NMS is triggered by the administration of dopamine receptor antagonists.^12^ The latter presents with akinesia, impaired consciousness, rhabdomyolysis, and raised body temperature and has also been reported in PD in relation to levodopa withdrawal.^13^ All these three entities share many clinical features including the insufficient response to dopaminergic drugs. NMS and akinetic crisis have even been regarded as the same entity by some authors.^14^ In our view, it is likely that DAWS, NMS and infection-related PD deterioration can be traced back to a common pathophysiological mechanism, presumably at the level of the dopamine receptor. Since Gerlach *et al.* identified infections as a further independent predictor for deterioration of PD patients during hospitalization,^7,8^ altered drug intake alone does certainly not account for all cases of motor deterioration during/after infections.

## Altered dopamine metabolism and receptor signaling

The best insights into the pathophysiology of motor deterioration during infections may come from the akinetic crisis, although this can be triggered not only by infections, but also by other factors such as abrupt drug withdrawal. In a retrospective analysis of 675 patients by Onofrj and Thomas,^4^ 26 patients had developed acute akinesia. In 17 of them, infections were identified as the precipitating factor. A hallmark of all cases was their prolonged unresponsiveness to antiparkinsonian drugs. Interestingly, l-DOPA serum levels were normal in all cases, including those with preceding infections. This notion excludes malabsorption as the explanation, and favors other disturbances in the dopamine metabolism or pathway as underlying causes.

One explanation for motor deterioration during systemic infections may be an altered transport of dopaminergic drugs through the blood–brain barrier (BBB). The permeability of the BBB is highly selective, and the transport of metabolites into the brain is regulated by active influx and efflux transporters. Likewise, l-DOPA and other dopaminergic drugs enter the brain through selective transporters,^15,16^ but can also be pumped out of the brain.^17^ The transporter functioning is in part dependent from electrolyte gradients across the cell membrane.^17^ Of note, disturbed electrolyte homeostasis may parallel systemic infections, and is concomitantly a precipitating factor for akinetic crisis.^13^ Inflammatory cytokines such as TNF-α released into the circulation during peripheral infections are also known to alter the activity and expression of endothelial transporters at the BBB.^18^ Interestingly, cytokines can upregulate the expression of efflux transporters, thus leading to an active return transport of drugs at the BBB.^19^

An altered presynaptic (re)uptake of l-DOPA and dopamine, respectively, and impaired packaging of neurotransmitters into vesicles, could be other explanations. It has been shown that cytokines reduce the expression of type 2 vesicular monoamine transporters, which are involved in transferring cytosolic dopamine into vesicles.^20^ The reuptake of released dopamine, in turn, is mediated by dopamine transporters (DAT). There is some evidence from *in vitro* studies that DAT expression is regulated by cytokines.^21^ In humans, the administration of IFN-α, an immunoregulatory cytokine, leads to an increased uptake and decreased turnover of ^18^F-DOPA in the striatum, and thus probably to a reduced release of dopamine at the nerve terminal.^22^ The reduced number of dopaminergic neurons in PD would suggest that this mechanism might be less relevant. However, astrocytes, which account for almost 50% of all brain cells, also express DAT, and they could potentially act as a relevant compartment for l-DOPA that might become inaccessible during infections.^23^

A further explanation could be that systemic infections lead to a downregulation of dopaminergic receptors on neurons, thus rendering them unresponsive to dopaminergic drugs, despite apparently delivering sufficiently high doses. It has been shown in non-human primates that chronic exposure to IFN-α decreases presynaptic D2 expression in the striatum.^24^ Outside the brain, proinflammatory cytokines such as TNF-α and IL-6 decrease the expression and impair the function of D1 receptors on renal cells.^25^ Similarly, neuronal glutaminergic AMPA receptor expression is also downregulated in response to TNF-α.^26^ However, little is known about the influence of cytokines on the expression and function of postsynaptic D2 receptor in the brain itself. Intriguingly, it has been shown that pertussis toxin can bind to a regulatory protein of the D2 receptor.^27^ Although pertussis might be a quite rare source of infections in PD patients, this observation implies that bacterial toxins may directly affect each of the aforementioned up- or downstream steps in the dopamine-signaling pathway.

In summary, there are many different steps in the dopamine metabolism, which can be potentially targeted by inflammatory cytokines during systemic infections.

### Enhanced neurodegeneration by peripheral inflammation

There is large body of evidence, mainly from animal studies, that dopaminergic degeneration and inflammatory processes are closely linked (reviewed in the study by Ferrari *et al*.^28^). Basically, peripheral infections may enhance neurodegeneration either through direct toxicity and noninflammatory effects of circulating bacterial toxins or through circulating cytokines that have been produced at the site of the inflammation. It has been shown that bacterial toxins are able to selectively damage dopaminergic neurons through inhibition of the ubiquitine–proteasome system, mitochondrial dysfunction, and increased oxidative stress and that dopaminergic degeneration was even increased, if mutations in gene PD genes such as alpha-synuclein or LRRK2 were present.^29,30^ In animal models, peripheral inflammation is also associated with an increased vulnerability to lipopolysaccharide induced degeneration within the substantia nigra.^31^ Circulating cytokines which are produced in the periphery during inflammatory processes (e.g., in urinary tract infections) can be transported through the BBB either via an increased BBB permeability, as seen in systemic inflammatory responses, or via increased active transport.^21^ In the brain, the presence of proinflammatory cytokines leads to the activation of quiescent microglia.^28^ Proinflammatory cytokines may also recruit monocytes, a cell population closely related to microglia, from the blood and make them invade the brain.^32^ In line with this, post-mortem analysis of brain tissues from PD patients have revealed the presence of activated microglia and proinflammatory cytokines in the substantia nigra.^33^ Activated microglia present cell surface receptors such as the class II major histocompatibility complex, and release proinflammatory cytokines and free radicals. These mechanisms, in turn, are considered to be closely related with neurodegeneration in PD.^28^ Intriguingly, PD patients with a known mutation in mitochondrial genes have a much higher risk of developing an akinetic crisis.^34^ This may reflect a higher susceptibility to oxidative stress and altered energy state during infections, at least in a particular subpopulation of PD patients. PD hence has some features in common with mitochondrial diseases (e.g., cytochrome-c oxidase deficiency), as in these conditions a deterioration and fluctuation of symptoms due to intercurrent infections and stress is also well recognized.^35^ Considering these findings from humans and animals, a further hypothesis could be that inflammatory processes located outside the brain may boost ongoing pre-existing neuroinflammatory processes in PD, thus leading to a clinical deterioration with incomplete recovery. The concept of enhanced neurodegeneration may well explain the observation that PD patients after suffering from an infection do not return to their previous best.

### Future perspective

To approach the afore-mentioned aspects, a prospective cohort study would be required. The cohort needs to be well characterized at study inclusion and followed-up over several years, so that the temporal course of PD progression is captured and any accelerated progression rate specifically related to infections can be distinguished from ‘background’ progression. Considering that an infection itself and any related deterioration of PD is an unpredictable and relatively infrequent event,^2^ the cohort has to be large enough to provide sufficient statistical power.

The issue of altered medication intake can be easily approached by monitoring drug intake by diaries, the use of electronic drug dispensers, or measuring 24 h drug plasma levels during the infection. Furthermore, precise recording of medication as a confounding factor is essential, as a clinical worsening might prompt practitioners or neurologists to adapt PD medications and the full extent of PD deterioration may hence be masked. The administration of antibiotics and/or anti-inflammatory drugs should also be considered as confounders, as they can influence the natural course of an infection, and as a neuroprotective effect of nonsteroidal antirheumatic drugs has been suggested.^36^ Changes in the dopamine metabolism and signaling can be approached by imaging studies, particularly by labeling dopamine receptors and transporters with radioactive tracers for PET or SPECT studies (i.e., (^123^I)-Ioflupane, (^18^F)-DOPA).^37^ Bioavailability of dopaminergic medications in the brain can be assessed by measuring CSF levels of the drugs or their metabolites or by comparing CSF and plasma drug levels. To quantify the extent of neurodegeneration associated with peripheral infection, a reliable biomarker for disease progression is required. This, however, brings us to one of the major challenges in PD research, as the search for the best biomarker fulfilling criteria such as good specificity and sensitivity, cost effectiveness, and standardization is still ongoing.^37^ CSF alpha-synuclein, tau and amyloid-beta have recently been proposed to correlate with motor progression,^38^ but whether they can capture short-term deterioration in PD remains to be seen. Magnetic resonance imaging techniques focusing on the structural integrity of gray and white matter or radiotracer techniques to label nigrostriatal projections may also be a feasible approach to quantify disease progression.^37^ Plasma and CSF levels of proinflammatory cytokines may serve as markers for the intensity of the inflammatory reaction. Moreover, CSF markers such as soluble CD14 and special PET tracers such as (^11^C)-(R)-PK11195 or (^11^C)vinpocetine may moreover allow tracking microglial activation.^39–41^ A more recently developed PET tracer (^11^C)-DED indicating astrocyte proliferation may also be a promising tool.^42^ An analysis of the metabolic profile in tissues with a high energy turnover (e.g., by using immunhistochemistry in muscle biopsies) can be useful to reveal alterations in the mitochondrial metabolism and increased levels of oxidative stress. Furthermore, genetic tests may allow identification of risk genes that are associated with an infection-related clinical deterioration in PD.

In order to reveal which findings are associated with prolonged or more severe clinical deterioration, a follow-up by repeating these tests is needed. Furthermore, a characterization of the infection, including location and type of infectious agent, might reveal whether some infection subtypes are more likely to result in PD worsening. A further challenge may be to clarify the relationship between motor deterioration and delirium in the framework of infections. Although similar pathophysiological mechanisms have also been proposed for delirium,^43^ the question arises whether they are different presentations of the same processes or their underlying pathophysiology is different. Taking into account the cohort size, study duration, and the proposed tests, the conduction of such a sophisticated study in this field will require huge personal, financial, and instrumental efforts.

## Conclusion

In theory, several different mechanisms may be involved in symptom deterioration of PD with systemic infections. Possible explanations are altered dopamine metabolism, insensitivity to dopamine at receptor level, enhancement of ongoing neuroinflammatory processes in PD, or altered drug intake. The available literature does not permit final conclusions on the underlying mechanisms, so that further studies in this field are therefore warranted. A long-term follow-up of PD patients including biomarkers and imaging assessments is probably the best approach to elucidate the relevant molecular pathways and to identify predictive risk factors.
